# Rapidly Destructive Coxopathy With Femoral Head Fracture That Is Similar to a Slipped Capital Femoral Epiphysis in an Elderly Woman: A Case Report

**DOI:** 10.7759/cureus.21997

**Published:** 2022-02-07

**Authors:** Takanori Miura, Hiroaki Kijima, Toshihito Ebina, Takayuki Tani, Naohisa Miyakoshi

**Affiliations:** 1 Orthopedic Surgery, Kakunodate General Hospital, Senboku, JPN; 2 Orthopedic Surgery, Akita University Graduate School of Medicine, Akita, JPN

**Keywords:** hip joint, femoral head fracture, rapidly destructive coxopathy, total hip arthroplasty: tha, cementless total hip arthroplasty

## Abstract

Slipped capital femoral epiphysis (SCFE) generally occurs in adolescents but rarely in adults. Rapidly destructive coxopathy (RDC) is characterized by rapid joint destruction, including disruption of the joint at the femoral head and acetabulum as well as reduction of the joint space, within six to 12 months. The mechanism of RDC is likely multifactorial but has not yet been identified. Moreover, there are no reports of displaced femoral head fractures similar to an SCFE associated with RDC. We report a rare case of RDC with femoral head fracture that is similar to SCFE in an 86-year-old woman. Although the exact cause of the femoral head fracture is unknown, it can develop into RDC. Awareness of orthopedic surgeons regarding this condition is crucial for appropriate treatment, by monitoring the presentation of symptoms and imaging/radiographic findings.

## Introduction

Slipped capital femoral epiphysis (SCFE) is defined as the displacement of the epiphysis relative to the metaphysis and shaft at the physis level, and it generally affects adolescents. An SCFE in adulthood is uncommon, with only a few reports of cases occurring with endocrine disorders [1]. Although a subchondral insufficiency fracture (SIF) of the femoral head is common in elderly women, there are only two cases of femoral head fracture similar to SCFE [2,3].

On the other hand, rapidly destructive coxopathy (RDC) is characterized by rapid joint destruction within six to 12 months without evidence of osteoarthritis, osteonecrosis of the femoral head, neuropathy, infection, or inflammatory disease [4-6]. The mechanism of RDC is likely multifactorial but has not been established. However, SIF seems to play an important role in the pathogenesis of RDC [4-8]. To the best of our knowledge, there are no reports of displaced femoral head fractures associated with RDC. We present a case of RDC with femoral head fracture that is similar to SCFE in an 86-year-old woman treated by applying total hip arthroplasty (THA).

## Case presentation

The patient was fully informed and provided consent to publish her data. An 86-year-old healthy woman presented to the hospital complaining of left hip pain in the absence of trauma, which had persisted for a month. She visited another hospital, but no abnormality was found on radiography. Subsequently, her symptoms worsened and she could not walk due to the pain, at which point she came to the hospital. She had no history of traumatic hip injury, rheumatoid arthritis, neuroarthropathy, collagen disease, seronegative arthritis, or hip injections (including steroids). She remained in a wheelchair due to hip pain. Upon physical examination, her left leg was shortened and externally rotated. She had tenderness at Scarpa’s triangle, and the external leg rotation was exacerbated during passive flexion of the hip.

**Figure 1:**
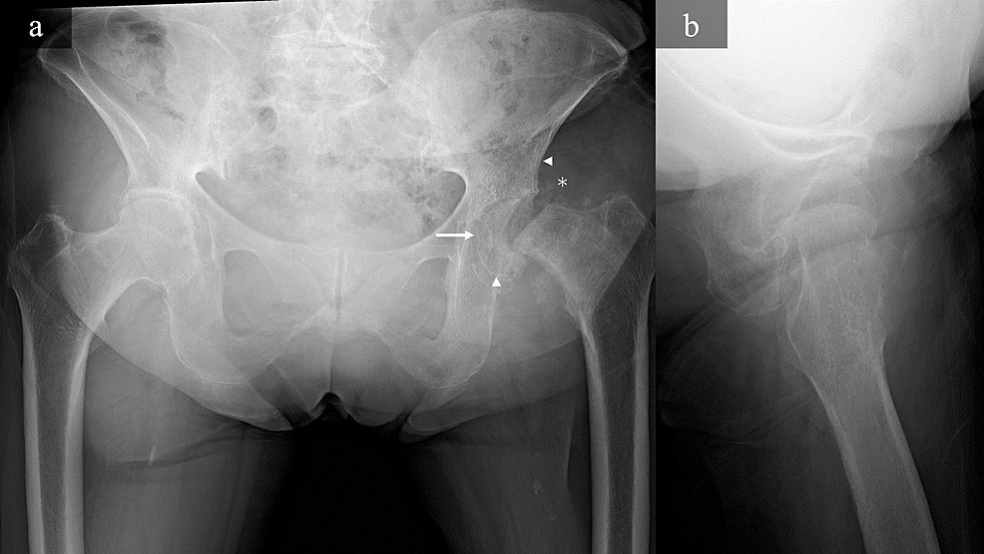
anteroposterior radiographs Anteroposterior (a) and lateral (b) radiographs show the femoral head fracture along the epiphyseal scar and displaced posteriorly (white arrow). The radiolucent area is seen at the femoral head and acetabulum (white arrowhead) and calcification around the hip joint (asterisk).

Blood tests showed an elevated white blood cell count of 9000/µL. Her C-reactive protein (CRP) was 0.62 mg/dL, and matrix metalloproteinase-3 (MMP-3) was elevated at 75.3 ng/mL. There was no history of secondary osteoporosis, and measurements of bone metabolism markers (bone alkaline phosphatase: 19.5 µg/L and tartrate-resistant acid phosphatase isoform 5b [TRACP-5b]: 796 mU/dL) suggested increased bone resorption. The antinuclear antibody and culture tests for joint fluid in the hip joint were negative. On anteroposterior radiographs, there was no evidence of developmental dysplasia of the hip; however, imaging revealed the femoral head was displaced at the epiphyseal scar to posterior-inferior, similar to SCFE, post-fracture changes, and joint destruction images (Figure 1).

**Figure 2:**
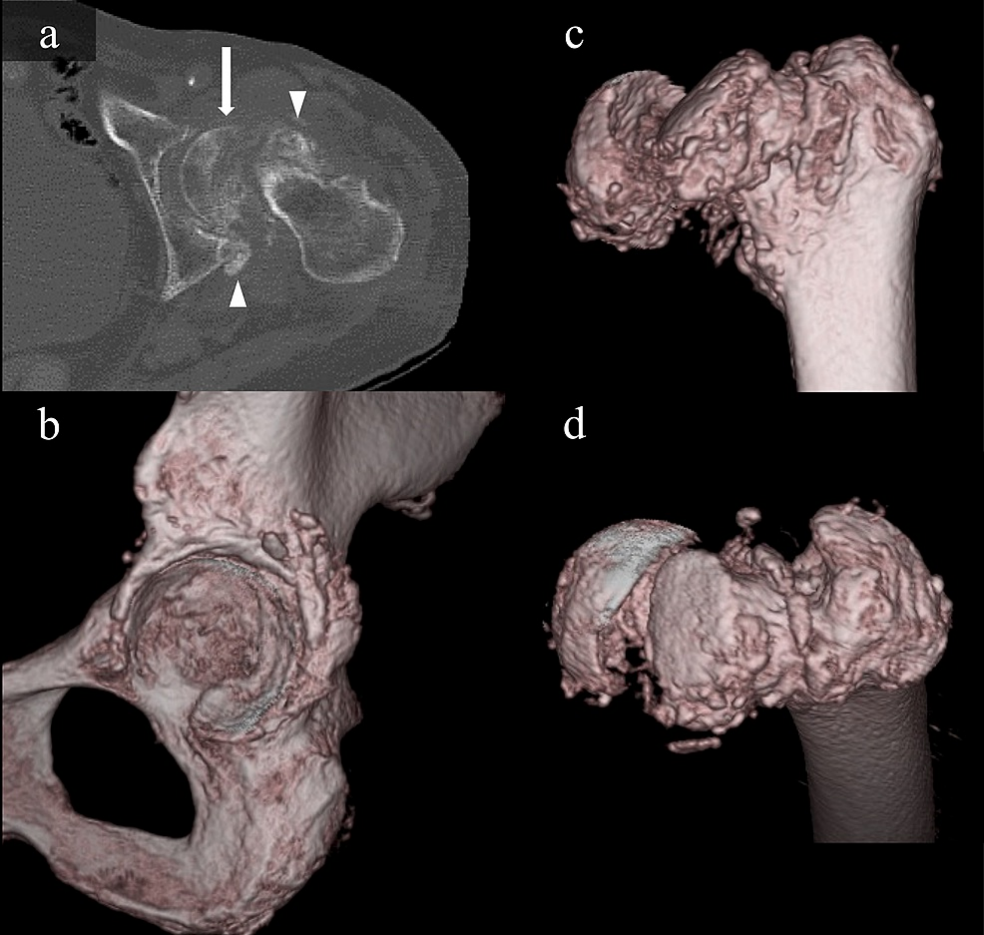
CT images of the acetabulum and femur (a) Axial CT images of the left hip show bone erosion in the displaced femoral head (white arrow) and calcification of the posterior acetabulum and anterior femur (white arrowhead). (b) 3D CT images of the acetabulum show mild destruction of the acetabulum and calcification around the posterior wall. (c), (d) 3D CT images showing the femoral head displaced posteriorly along the epiphyseal scar.

On 3D CT images, the posterior displacement of the femoral head was more apparent (Figure 2).

**Figure 3:**
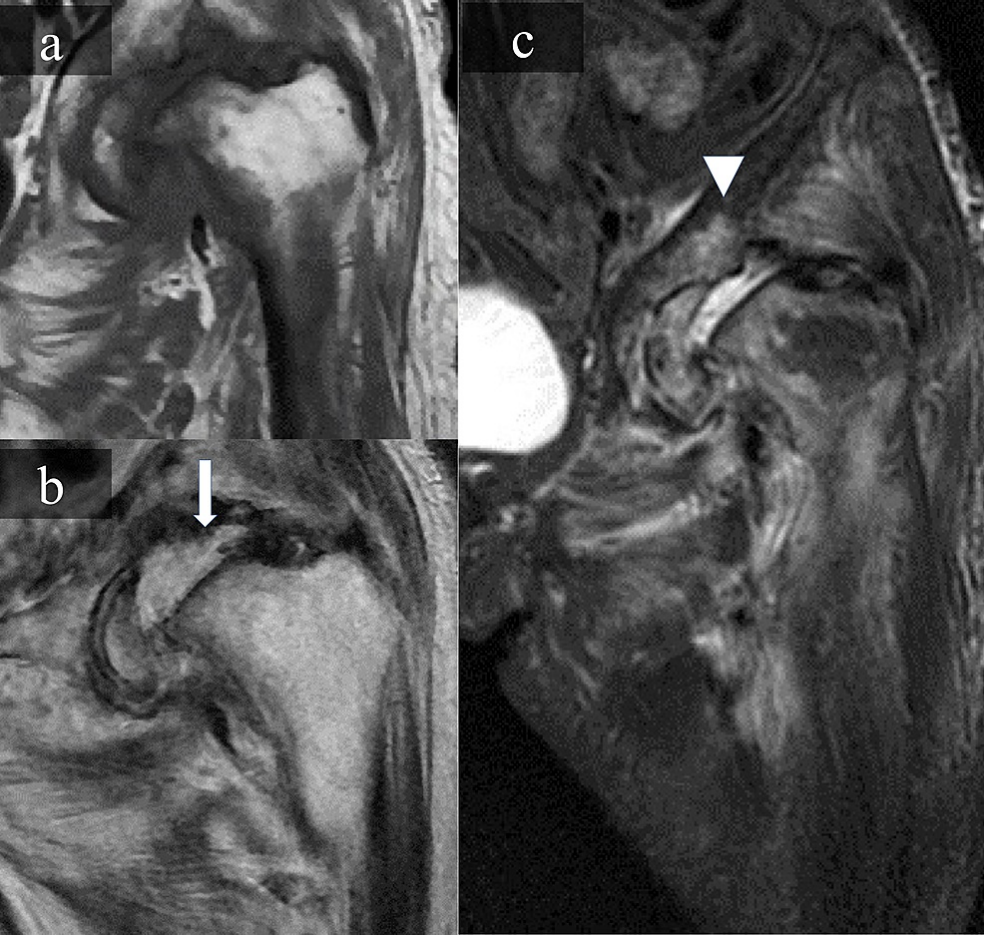
Magnetic resonance images of the hip Magnetic resonance images showed low signal intensity in femoral head on T1-weighted imaging (a), Joint effusion (white arrow) on T2- weighted imaging (b), high signal intensity in the acetabulum (white arrowhead), hip muscles on short tau inversion recovery imaging (c).

MRI showed joint effusion was apparent on T2-weighted sequences and high signal intensity areas at the acetabulum, femoral head, and hip muscles on short tau inversion recovery sequences (Figure 3).

**Figure 4:**
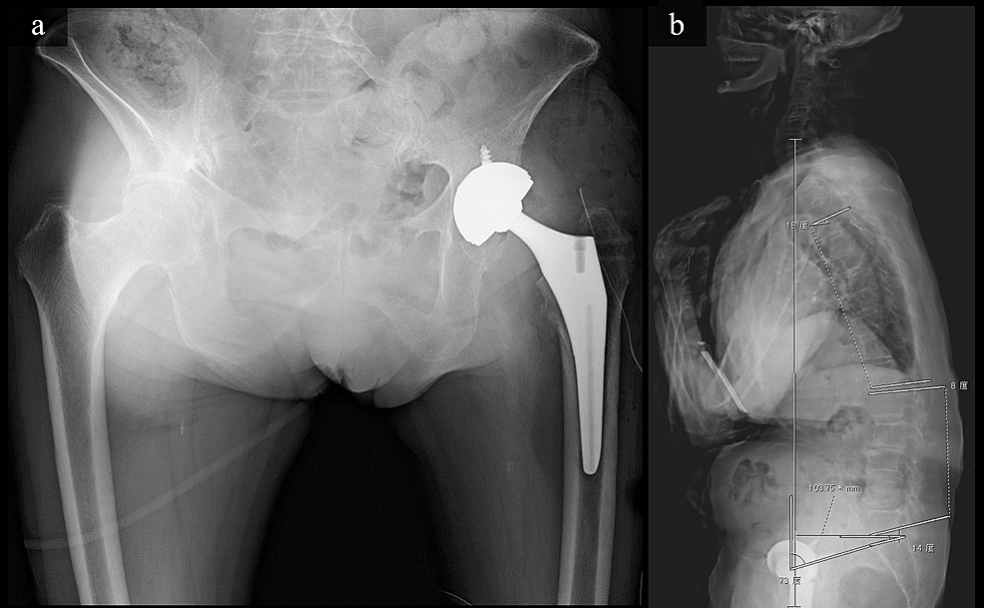
postoperative radiograph (a) Anteroposterior radiograph after total hip arthroplasty. (b) Lateral radiograph of the whole spine in the upright position post-surgery.

Bone mineral density (BMD) examination using dual-energy X-ray absorptiometry showed a lower bone mass and density expected for her age. Her lumbar spine BMD was 0.766 g/cm2, with a corresponding T-score of -2.0. BMD of the right hip was 0.583 g/cm2, corresponding to a T-score of -2.3. The patient was diagnosed with RDC, and THA through lateral approach was performed. Surgical findings showed that the femoral head was completely displaced but the cartilage was relatively preserved, the synovium was thickened, and a callus was seen around the femur neck. Throughout the acetabulum, the cartilage was destructed, bone quality was poor, and the stock was weak. Hence, we cautiously avoided penetrating the medial wall while reaming. The bone quality of the proximal femur was relatively good. We used a cementless cup with added screw fixation in combination with a tapered wedge-type cementless stem (Stryker, Kalamazoo, MI, USA). Postoperative radiographs showed satisfactory component position. The post-operative standing lateral radiographs of the entire spine showed a decreased lumbar lordosis to 8°, an increased pelvic tilt to 73°, and an anterior trunk tilt with a sagittal vertical axis of 103 mm, thereby indicating sagittal imbalance position (Figure 4).

**Figure 5:**
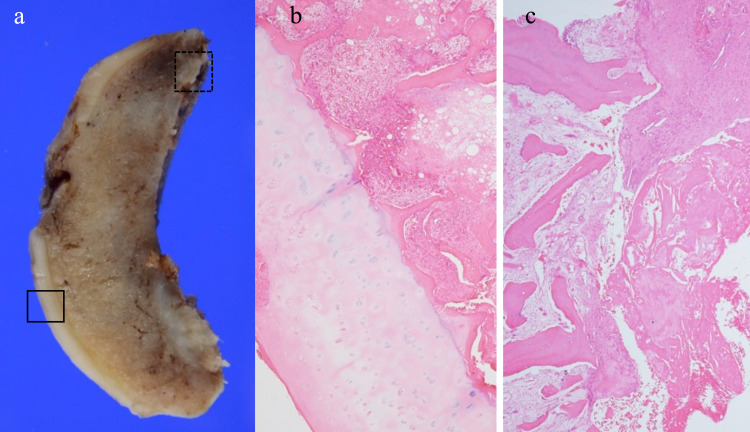
Pathological examination of the femoral head (a) Gross appearance of the resected femoral head. (b) Photomicrograph of the area bounded by the black rectangle in (a), (c) Photomicrograph of the area bounded by the black dotted rectangle in (a) (hematoxylin and eosin ×20).

She was discharged, walking with a cane, and without any postoperative complications. Pathological examination of the femoral head showed no articular cartilage degeneration or necrosis of the subchondral bone in the non-weight-bearing area. However, post-fracture changes, such as callus and fibrous tissue formation and focal osteonecrosis, were observed at the epiphyseal scar area (Figure 5). Finally, we diagnosed the patient with RDC secondary to a fragility femoral head fracture.

## Discussion

RDC describes the rapid and aggressive destruction of the unilateral hip joint, defined as chondrolysis >2 mm or 50% joint space narrowing in one year [5]. Therefore, it is necessary to confirm the progression of hip arthropathy using X-ray imaging tests at diagnosis. In some cases, the diagnosis is difficult based on imaging findings alone [9]. The clinical-radiological diagnosis criteria include the absence or reduction of osteophytes, as well as the presence of geodes in the femoral head and acetabulum [10]. In the early stages of RDC, narrowing of the joint space is usually found in the superior lateral aspects on the radiograph, and bone marrow edema is observed in the femoral head and acetabulum. Additionally, joint effusion, synovitis, and extracapsular edema are evident on MRI images [11,12]. A systematic review revealed flattening of the femoral head in the weight-bearing area, absence of articular cartilage, and evidence of some destruction of the subchondral bone on radiographs of all RDC patients [9]. Blood tests showed that inflammatory responses, such as an elevated erythrocyte sedimentation rate (ESR), CRP, MMP3, and serum TRACP-5b, were significantly higher, which may be due to osteoclast cell activation in the hip joints [8,13,14]. In addition, for the diagnosis of RDC, the specificity of TRACP5b (with a cut-off value of 623 mU/dL) was 85.3%, which is useful as an RDC marker [8].

Concerning the pathogenesis of RDC, SIF has been identified as one of the primary causes of RDC [6]. However, to our knowledge, there are only two cases of femoral head fractures of the epiphyseal scar that are similar to SCFE in the elderly [2,3]. De Silva et al. reported after internal fixation using a dynamic hip screw for fracture of a displaced basi-cervical femoral fracture. A femoral head fracture similar to SCFE occurred distal to the screw tip, requiring hemiarthroplasty [2]. Baba et al. reported a case of femoral head fracture similar to SCFE, which occurred several years after conservative treatment for SIF and resulted in THA [3]. In this case, the exact onset time and cause of the femoral head fracture are unknown because the patient did not experience a traumatic event, and the hip destruction had progressed significantly by the time the patient visited our hospital. In addition, although changes in radiographs such as progressive narrowing of the hip joint space or femoral head destruction over time are necessary for the diagnosis of RDC, we comprehensively diagnosed the patient with RDC based on imaging findings. Including the narrowing of the joint space, particularly in the superior lateral area in the radiographs, bone marrow, extracapsular edema, and joint effusion on MRI. Additional testing revealed an elevated CRP, MMP-3 and TRACP-5b in blood tests and histopathological findings. Increased abnormal shear stress along the epiphyseal scar has been reported to cause SCFE-like femoral head fracture [2,3]. In this case, we speculate that femoral head fracture occurred by the same mechanism, subsequently progressing to RDC due to synovitis and osteoclast activation. This disease progression is similar to that of SIF to RDC. In addition, if the femoral head fracture had been diagnosed at an early stage, it could have been treated with hemiarthroplasty. Therefore, detailed examination, including MRI and careful follow-up, are necessary in cases of hip pain in elderly patients.

## Conclusions

In conclusion, femoral head lesions without any obvious causes in the elderly should be differentiated from osteonecrosis of the femoral head, infection, and SIF. Even in the absence of a history of trauma, there are cases of fractures of the femoral head at the epiphyseal scar. Furthermore, such fractures can develop into RDC due to inappropriate or insufficient treatment. Therefore, awareness of orthopedic surgeons regarding this condition is crucial in providing appropriate treatment, by thoroughly monitoring the development of symptoms and imaging findings, particularly among elderly patients.
